# Genome mining and biosynthetic pathways of marine-derived fungal bioactive natural products

**DOI:** 10.3389/fmicb.2024.1520446

**Published:** 2024-12-12

**Authors:** Caihua Han, Anjing Song, Yueying He, Liu Yang, Litong Chen, Wei Dai, Qilin Wu, Siwen Yuan

**Affiliations:** ^1^School of Bioengineering, Zunyi Medical University, Zhuhai, China; ^2^Center of Ocean Expedition, School of Atmospheric Science, Sun Yat-sen University, Zhuhai, China; ^3^Teaching and Experimental Center, Guangdong Pharmaceutical University, Guangzhou, China

**Keywords:** marine fungi, marine natural products, biosynthetic gene clusters, genome mining, biosynthesis

## Abstract

Marine fungal natural products (MFNPs) are a vital source of pharmaceuticals, primarily synthesized by relevant biosynthetic gene clusters (BGCs). However, many of these BGCs remain silent under standard laboratory culture conditions, delaying the development of novel drugs from MFNPs to some extent. This review highlights recent efforts in genome mining and biosynthetic pathways of bioactive natural products from marine fungi, focusing on methods such as bioinformatics analysis, gene knockout, and heterologous expression to identify relevant BGCs and elucidate the biosynthetic pathways and enzyme functions of MFNPs. The research efforts presented in this review provide essential insights for future gene-guided mining and biosynthetic pathway analysis in MFNPs.

## 1 Introduction

The ocean, often regarded as the cradle of life, hosts a rich diversity of species within unique ecological niches, fostering distinctive marine organisms that have generated a plethora of structurally novel and biologically active metabolites essential for new drug development. By the end of 2022, over 37,542 new marine natural products (MNPs) have been documented, predominantly comprising polyketides, terpenoids, alkaloids, and non-ribosomal peptides (Carroll et al., 2022; Carroll et al., 2019, Blunt et al., 2018, Carroll et al., 2020, Carroll et al., 2021, Carroll et al., 2024, Carroll et al., 2023). Up to 15 MNP-derived pharmaceuticals have been approved for market, including cytarabine (Cytosar-U), vidarabine (Vira-A), and eribulin mesylate (Halaven) from sponges, ziconotide (Prialt) from the venom of the pacific fish-hunting marine mollusk *Conus magus*, omega-3-acid ethyl esters (Lovaza and Vascepa) from fish body oils, trabectedin (Yondelis), plitidepsin (Aplidin), and lurbinectedin (Zepzelca) from sea squirts, and brentuximab vedotin (Adcetris), enfortumab vedotin (Padcev), polatuzumab vedotin (Polivy), belantamab mafodotin (Blenrep) from *Dolabella auricularia* and *Symploca* sp (Papon et al., 2022). Additionally, 33 MNP-derived pharmaceutical were undergoing clinical trials, with 5 in Phase III, 12 in Phase II, and 16 in Phase I stages (Patridge et al., 2016). These findings underscore the pivotal role of marine natural products in pharmaceutical development.

**FIGURE 1:**
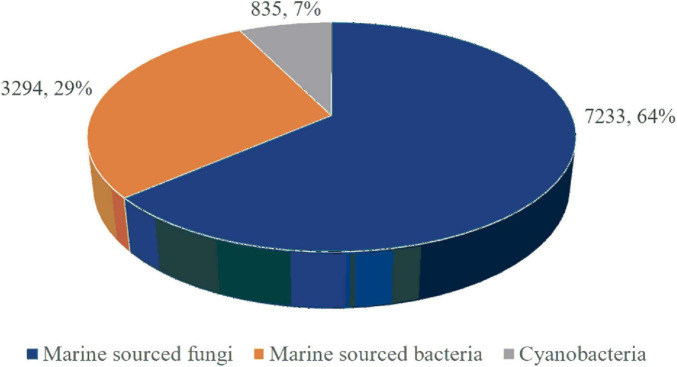
The proportions of MNPs from marine microorganisms.

Marine microorganisms, thriving in unique oceanic environments, possess specialized metabolic and defensive mechanisms, thereby facilitating the production of structurally novel bioactive MNPs, making marine microorganisms as crucial sources for new MNPs. Approximately 11,362 new MNPs have been discovered from marine microorganisms, constituting 30% of all known marine natural products. Among these, 63.8% (7,233) originate from marine fungi, 28.9% (3,294) from bacteria, and 7.3% (835) from cyanobacteria (Figure 1; Blunt et al., 2018, Carroll et al., 2019, Banerjee et al., 2022, Voser et al., 2022, Carroll et al., 2022, Carroll et al., 2020, Carroll et al., 2023, Carroll et al., 2024). Thus, marine fungi emerge as the predominant source of marine microbial natural products.

MFNPs represent a significant source of marine microbial natural products. However, most remain underdeveloped, with only a small fraction documented (Costantini, 2020, Wei et al., 2021a). This is largely due to the unexplored BGCs responsible for MFNPs production, indicating that activating these BGCs holds substantial potential for advancing drug discovery. Therefore, understanding and activating these silent BGCs is essential for advancing novel drug development, as well as for exploring biosynthetic pathways and identifying associated enzymes to enhance MFNPs development and discover new pharmaceuticals. Currently, various advanced genome mining strategies, including heterologous expression in model fungi (Biggins et al., 2011, Yuan et al., 2022a), targeted inactivation of key genes (Wei et al., 2021a,Ning et al., 2024), one-strain-many-compounds (OSMAC) (Scherlach and Hertweck, 2006, Scherlach et al., 2010), chemical epigenetic modifications (Zheng et al., 2017, Fan et al., 2017), and overexpression of transcription factor (Zhang et al., 2018), are widely employed to activate silent BGCs. These efficient methodologies facilitate the targeted discovery of bioactive compounds, addressing the challenges of randomness and inefficiency traditionally associated with natural product exploration. This review consolidates progress in the genome mining and biosynthesis of polyketides, terpenes, alkaloids, and cyclic peptides from marine fungi, providing insights for the future BGC-guided discovery of MFNPs (Table 1).

**TABLE 1:** The summarizing of MFNPs.

Compounds	Bioactivity	Source	Cluster	Genome mining methods	References
Flavoglaucin (HR-PKS)	Anti-inflammatory, anticancer	*Eurotium cristatum*, *Eurotium repens*, *Eurotium herbariorum*	*fog*	Bioinformatic analysis, heterologous expression	Zhang P. et al., 2019; Smetanina et al., 2007; Miyake et al., 2009; Nies et al., 2020
Griseofulvin (NR-PKS)	Antifungal, anticancer	*Penicillium griseofulvum* Dierckx	*gsf*	Gene knockout, heterologous expression	Oxford et al., 1939; De Carli and Larizza, 1988; Chooi et al., 2010; Cacho et al., 2013; Lane et al., 2002; Harris et al., 1976
Sorbicillinoids (PKS)	Anti-inflammatory, anticancer, antibacterial activity, Anti-HIV	*Trichoderma reesei*4670, *Trichoderma reesei*(HN-2016-018), *Stagonospora* sp. SYSU-MS7888, *Penicillium* sp. SCSIO06868	*sor*	Bioinformatic analysis, gene knockout	Harned and Volp, 2011; Andrade et al., 1992; Zhang P. et al., 2019; Rehman et al., 2020; Chen et al., 2022b; Pang et al., 2022
Monodictyphenone (NR-PKS)	Cytotoxicity	*Monodictys putredinis*, *Diaporthe* sp. SYSU-MS4722	*mdp*	Gene knockout	Krick et al., 2007; Chen et al., 2022a; Chiang et al., 2010
Epicospirocins (NR-PKS)	Cytotoxicity, antimicrobial activity	*Aspergillus micronesiensis*	*esp*	Gene knockout	Luyen et al., 2019; Zhu et al., 2020
Chrysoxanthones (NR-PKS)	Antibacterial activity	*Penicillium chrysogenum* HLS111		Bioinformatic analysis	Zhen et al., 2018
Phomoxanthone A (NR-PKS)	Cytotoxicity, antimicrobial activity, antifungal	*Diaporthe* sp. SYSU-MS4722	*pho*	Heterologous expression Gene knockout	Yuan et al., 2022b; Chen et al., 2022a
Amphichopyrones (PKS)	Anti-inflammatory	*Amphichorda felina* SYSU-MS7908	*Amp*	Heterologous expression	Yuan et al., 2022a
Penilactones (PKS)	NF-κB inhibitory activity	*Penicillium crustosum* PRB-2	*Cla* *tra*	Heterologous expression	Wu et al., 2012; Dai et al., 2022; Fan et al., 2019; Fan et al., 2020
Alternapyrone G (NR-PKS)	Anti-inflammatory Neuroprotective effect	*Arthrinium arundinis*	*alt*′	Heterologous expression	Hu et al., 2024; Hu et al., 2024
Chevalone (Terpenes)	Antibacterial activity, anticancer	*Aspergillus milianensis* KUFA 0013	*Cle*	Heterologous expression	Prompanya et al., 2014; Xiao et al., 2022
Ophiobolins (Terpenes)	Anticancer	*Aspergillus ustus* 094102	*Obl*	Gene knockout Gene replacement Heterologous expression	Zhang et al., 2012; Tian et al., 2017; Yan et al., 2022; Chai et al., 2016
Aspergildienes, Aspergilols (Terpenes)	Cytotoxicity Anticancer	*Aspergillus ustus* 094102	__	Heterologous expression	Guo et al., 2021
Spiromaterpenes (Terpenes)	Anti-inflammatory	*Spiromastix* sp.	*spt*	Heterologous expression	Guo et al., 2021; Burkhardt et al., 2016
Asperaculin A (Terpenes)	__	*Aspergillus aculeatus* CRI323-04	*aspe*	Heterologous expression	Ingavat et al., 2011; Das and Chakraborty, 2016; Wei et al., 2021b; Zeng et al., 2019; George et al., 2021
Talaronoids (Terpenes)	Butyrylcholinesterase (BChE) inhibitory activity	*Aspergillus flavipes* CNL-338	*tnd*	Heterologous expression	Zhang et al., 2020
Ascochlorin (Meroterpenoids)	Antibacterial activity, Antitumor Antiviral activity, anti-inflammatory	*Acremonium Sclerotigenum*, *Stilbella fimetaria*	*asc*	Transcriptome analysis Gene knockout Heterologous expression	Subko et al., 2021; Araki et al., 2019
Chrodrimanins (Meroterpenoids)	Inhibit protein tyrosine phosphatase 1B (PTP1B)	*Talaromyces* sp. CX11	*cdm*	Heterologous expression	Cao et al., 2019; Bai et al., 2018
Verruculides (Meroterpenoids)	Inhibit protein tyrosine phosphatase 1B (PTP1B)	*Talaromyces purpureogenus*	*cdm*	Heterologous expression	Cao et al., 2020; Bai et al., 2018
Talaromyides (Meroterpenoids)	Antiviral activity	*Penicillium* sp. SCS-KFD09	*tlx*	Heterologous expression	Kong et al., 2017; Li et al., 2021
Gliotoxin (NRPS)	Antibacterial activity, cytotoxic activity	*Neosartorya pseudofischeri*	*gli*	Bioinformatics analysis Gene knockout	Liang et al., 2014; Scharf et al., 2016; Scharf et al., 2014; Gardiner and Howlett, 2005; Balibar and Walsh, 2006; Chang et al., 2013; Davis et al., 2011; Scharf et al., 2011
Oxopyrrolidines (NRPS)	Antibacterial activity, antifungal, cytotoxicity	*Penicillium oxalicum* MEFC104	*opd*	Bioinformatic analysis Gene knockout	Li et al., 2022; Bergmann et al., 2007
Psychrophilins (NRPS)	Anticancer	*Aspergillus versicolor* ZLN-60	*psy*	Gene knockout	Ebada et al., 2014; Peng et al., 2014; Zhao et al., 2016
Asperalins (NRPS)	Insecticidal, antibacterial Antifungal, antitumor, antiviral activity	*Aspergillus alabamensis* SYSU-6778	*apl*	Heterologous expression	Hu et al., 2023; Zeng et al., 2024
Isoindolinones (Alkaloids)	Fibrinolytic effects	*Stachybotrys longispora* FG216	*stb*	Bioinformatics analysis	Shinohara et al., 1996; Hu et al., 2001; Hasegawa et al., 2010; Koide et al., 2012; Yin et al., 2017; Yin et al., 2017

In the compounds column, the text within the parentheses represents the refined classification.

## 2 Marine fungi-derived natural products

### 2.1 Polyketides

#### 2.1.1 Flavoglaucin

Flavoglaucin (**1**), dihydroauroglaucin (**2**) and isodihydroauroglaucin (**3**), are derived from various marine fungi, including those derived from sea lilies *Eurotium cristatum* (Zhang P. P. et al., 2019), the sponge-derived fungus *Eurotium repens* (Smetanina et al., 2007), and the bonito-derived fungus *Eurotium herbariorum* (Miyake et al., 2009). Compounds **1**, **2** and **3** have exhibited significant inhibitory properties on lipopolysaccharide (LPS)-activated NO production, with IC_50_ values of 0.46, 3.30, and 0.46 μM, respectively. Additionally, compound **1** has demonstrated cytotoxic effects on HepG2 (liver cancer) and HeLa (cervical cancer) human cancer cell lines, with IC_50_ values of 41.48 ± 3.52 and 33.60 ± 1.32 μM, respectively (Zhang P. P. et al., 2019).

**FIGURE 2:**
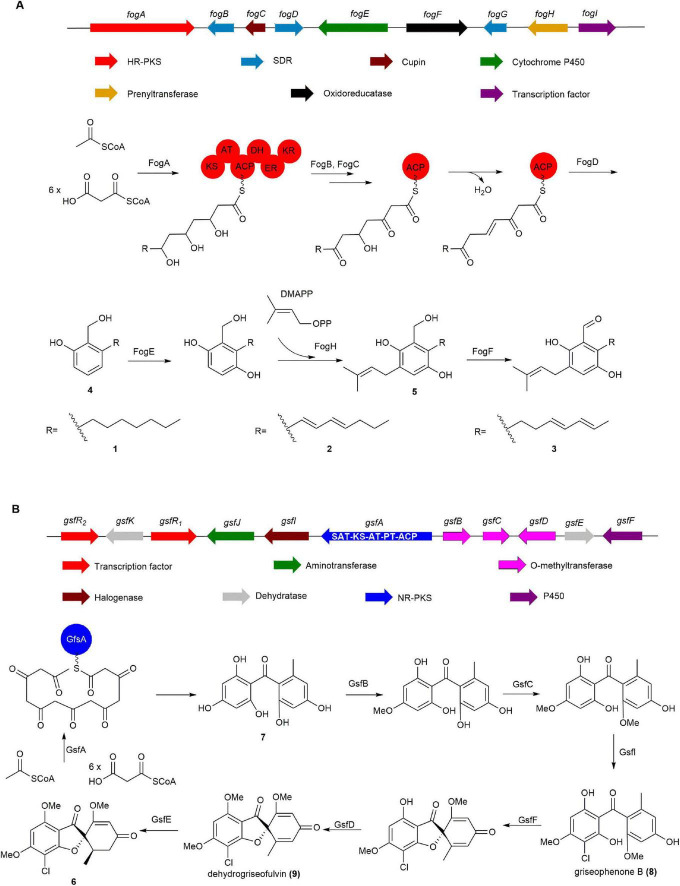
The BGCs and biosynthetic pathways of **(A)** flavoglaucin (**1**) and **(B)** griseofulvin (**6**). SAT, starter unit:ACP transacylase; KS, ketosynthase; AT, acyltransferase; ACP, acyl carrier protein; PT, product template.

The BGC *fog*, responsible for the production of **1** and its derivatives, was identified by Li group from *Aspergillus ruber* through bioinformatic analysis (Nies et al., 2020). It was discovered that *fog* shares over 40% homology with the BGCs of trichoxide and sordarial, both analogs of **1**, suggesting that its potential to produce salicylaldehyde natural products. The co-expression of highly reducing polyketide synthase (HR-PKS) (*fogA*), SDR (*fogBD*), and Cupin (*fogC*) of from *fog* in *Aspergillus nidulans* LO8030 led to the isolation of **4**. Subsequent introduction of the prenyltransferase FogH and cytochrome P450 FogE led to the formation of isoprenylated **5**. Eventually, feeding experiments demonstrated that **5** undergoes catalysis by the oxidoreductase FogF to produce **1** and its derivatives (Figure 2A; Nies et al., 2020).

#### 2.1.2 Griseofulvin

Griseofulvin (**6**), an antifungal drug that disrupts fungal cell mitosis, is derived from *Penicillium griseofulvum* Dierckx, which was identified in the deep-sea region of the Indian Ocean in 1939 (Oxford et al., 1939, De Carli and Larizza, 1988). Compound **6** used to treat superficial infections, exhibits a fungistatic effect on various types of dermatophytes, including trichophyton, microsporum, achorion, and epidermophyton species (Vardanyan and Hruby, 2006). Furthermore, **6** possesses the ability to disrupt mitotic spindles and potentially inhibit centrosomal clustering, which are properties that hold promise for cancer treatment (Tsunematsu et al., 2020, Panda et al., 2005, Rebacz et al., 2007). Additionally, **6** has demonstrated significant apoptotic activity in diverse human and murine myeloma and lymphoma cell lines, as well as in human primary cells (Kim et al., 2011).

Tang group confirmed the BGC *gsf* of **6** through gene knockout experiments and successfully elucidated the biosynthesis of **6** by *in vitro* reconstitution of each enzyme in the *gsf* cluster. Gene deletions confirmed that non-reducing PKS (NR-PKS) gsfA is essential for the biosynthesis of **6**, playing a pivotal role in catalyzing the formation of benzophenone **7**. Diverging from conventional NR-PKS enzymes, GfsA does not incorporate a TE domain, thereby indicating that the release of **7** is likely mediated by its PT domain (Chooi et al., 2010, Cacho et al., 2013). Then **7** undergoes modification by two methoxyltransferases, GsfB and GsfC, and chlorination by the halogenating enzyme GsfI, resulting in the formation of griseophenone B (**8**). Subsequently, P450 enzyme GsfF and methoxyltransferase GsfD catalyze the formation of spirocyclic structures and subsequent methylation to yield dehydrogriseofulvin (**9**). Finally, GsfE reduces the C_2_–C_3_ double bond to a single bond, thereby producing the final product **6** (Figure 2B; Lane et al., 2002, Harris et al., 1976).

#### 2.1.3 Sorbicillinoids

Sorbicillinoids are a family of hexaketide metabolites characterized by a distinctive sorbyl side chain residue, first isolated as impurities in penicillin in 1948 (Harned and Volp, 2011, Andrade et al., 1992). Sorbicillinoids natural products are widely present in various marine fungi, such as sponge derived fungi *Trichoderma reesei*4670 (Zhang P. et al., 2019), *Trichoderma reesei* (HN-2016-018) (Rehman et al., 2020), *Stagonospora* sp. SYSU-MS7888 (Chen et al., 2022b), and *Penicillium* sp. SCSIO06868 (Pang et al., 2022), and exhibit significant anti-inflammatory (Pang et al., 2022, Chen et al., 2022b,Zhang P. et al., 2019, Zhao et al., 2017), anticancer (Rehman et al., 2020), antibacterial (Warr et al., 1996), and anti-HIV activities (Zhao et al., 2017).

**FIGURE 3:**
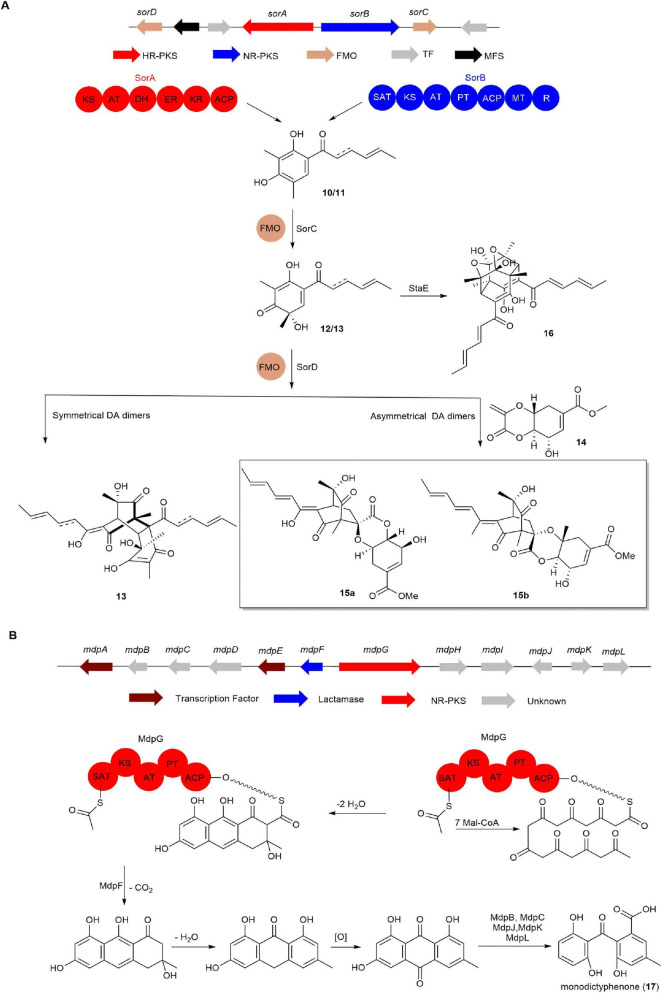
The BGCs and biosynthetic pathways of **(A)** sorbicillinoids and **(B)** monodictyphenone (**17**).

In 2014, the FAD-dependent monooxygenase gene *sorC* from *Penicillium chrysogenum* E01-10/3 was expressed in *Escherichia coli* by Cox group. SorC effectively catalyzed the oxidative dearomatization of sorbicillin (**10**) and dihydrosorbicillin (**11**), producing sorbicillinol (**12**) and dihydrosorbicillinol (**13**). Combining bioinformatic analysis with experimental data, the BGC responsible for sorbicillinoids was preliminarily confirmed (Fahad et al., 2014). Mach-Aigner group conducted further investigation into the biosynthetic pathway of **12** in *T. reesei* through gene knockout and *in vitro* enzyme catalysis. They discovered that knocking out the flavin-dependent monooxygenase gene *sorD* resulted in a significant increase in the amount of reduced branched double bonds in **12**. This led to the inference that sorD primarily catalyzes the formation of branched double bonds at positions 2 and 3 in **12** (Derntl et al., 2017). However, subsequent research by the Cox group revealed that sorD also possesses dimerization activity. It can catalyze the Diels-Alder reaction of **12** to produce homodimerization product **13**, as well as catalyze the Diels-Alder reaction between **12** and **14** to produce heterodimerization product **15**. This marks the first report of sorD functioning as a dimerase that catalyzes intermolecular Diels-Alder reactions (Figure 3A; Kahlert et al., 2020). Trichodimerol (**16**) is a unique cage-like dimeric sorbicillinoid pigment commonly isolated from many marine fungi. In 2023, Gao group reported that a major facilitator superfamily transporter (StaE) from marine-derived fungus *Stagonospora* sp. SYSU-MS7888 is involved in the formation of **16** (Figure 3A; Ren et al., 2023).

#### 2.1.4 Monodictyphenone

Monodictyphenone (**17**) is benzophenone derivatives with significant biological activities. Compound **17**, isolated from the marine algicolous fungus *Monodictys putredinis* (Krick et al., 2007) and the ascidian-derived fungus *Diaporthe* sp. SYSU-MS4722 (Chen et al., 2022a), serves as common precursor for various complex natural products biosynthesis with anthraquinone and xanthone structures (Simpson, 2012, Neubauer et al., 2016, Sanchez et al., 2011, Schatzle et al., 2012, Matsuda et al., 2018, Griffiths et al., 2016, Wei and Matsuda, 2020, Wei et al., 2021c).

Compound **17** is a common precursor in the biosynthesis of anthraquinone and xanthone. The BGC of **17** was characterized by the Oakley group. They discovered that knocking out the *cclA* gene, responsible for histone H3K4 methylation, successfully led to the identification of **17** in *A. nidulans*. Further, knocking out the NR-PKS gene *mdpG* in the *cclA*-inactivated *A. nidulans* strain resulted in the complete disappearance of **17** in the mutant strain, thereby identifying the *mdp* BGC of **17** (Chiang et al., 2010). When the two transcription factor genes, *mdpA* and *mdpE*, in the *mdp* cluster were knocked out, the corresponding mutant strains showed a significant decrease in **17** production. This indicates that the transcription factors MdpA and MdpE play a positive regulatory role in the production of **17** in *A. nidulans*. Additionally, knocking out the β-lactamase gene *mdpF* resulted in the complete disappearance of **17**, demonstrating that the β-lactamase MdpF is essential for the early biosynthesis of **17**. Subsequently, the biosynthetic pathway of **17** was inferred through bioinformatics analysis (Figure 3B; Chiang et al., 2010).

#### 2.1.5 Epicospirocins

Epicospirocins are natural products of the dibenzospirone class with various pharmacological activities, primarily derived from marine fungi. For instance, aspermicrones B (**18**) and C (**19**), isolated from the seaweed-derived endophytic fungus *Aspergillus micronesiensis*, show significant bioactivities. Compound **18** exhibited a selective cytotoxic effect toward the HepG2 cell line (IC_50_ = 9.9 μM), and both **18** and **19** displayed antimicrobial activity against *Staphylococcus aureus* (MIC = 123.2 μM for each compound) (Luyen et al., 2019).

**FIGURE 4:**
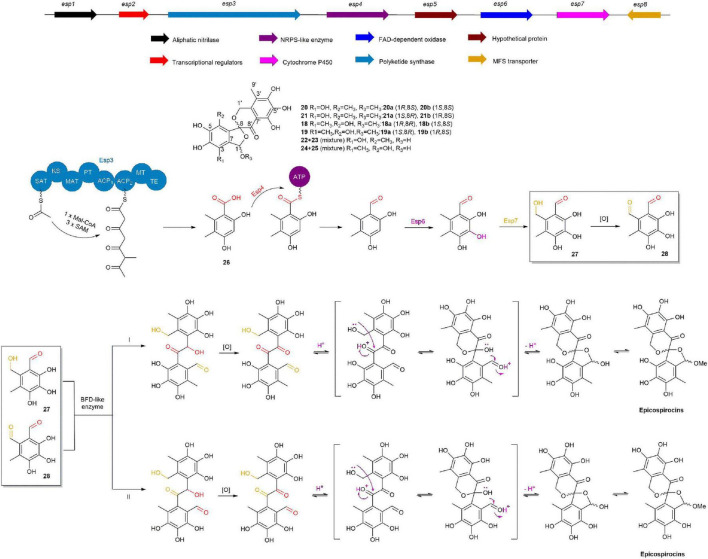
The BGC and biosynthetic pathway of epicospirocins.

In 2020, the Liu group used molecular network technology to uncover two pairs of dibenzospiroketal racemates, (±)-epicospirocin A (**20a**/**20b**) and (±)-1-epi-epicospirocin A (**21a**/**21b**), along with two (+)-enantiomers of aspermicrones, ent-aspermicrone B (**18b**) and ent-aspermicrone C (**19b**), and two hemiacetal epimeric mixtures, epicospirocin B/1-epi-epicospirocin B (**22**/**23**) and epicospirocin C/1-epi-epicospirocin C (**24**/**25**) from the fungus *Epicoccum nigrum* 09116. Through gene function annotation, gene knockout, and mass spectrometry analysis, they identified the BGC of epicospirocins and proposed its biosynthetic pathway. Knocking out the *pks* gene in the Δ*esp3* mutant strain resulted in the complete absence of epicospirocins and their analogs, indicating that Esp3 is crucial for the biosynthesis of the 5-methylorsellinic acid (**26**) skeleton. Subsequently, construction of a Δ*esp4* mutant strain led to the accumulation of a significant amount of **26**, demonstrating that Esp4 recognizes **26** and reduces its carboxyl group to an aldehyde group in **26**. Esp6 and Esp7 were found to be primarily responsible for the sequential hydroxylation of the benzene ring and methyl group, leading to the formation of **27** and **28**. Ultimately, **27** and **28** are converted into epicospirocins through the actions of multiple post-modifying enzymes (Figure 4; Zhu et al., 2020).

#### 2.1.6 Chrysoxanthones

Using an epigenetic strategy, three heterodimeric tetrahydroxanthone–chromanone lactones, chrysoxanthones A–C (**29**–**31**), were discovered from the sponge-associated *Penicillium chrysogenum* HLS111 by treating it with the histone deacetylase inhibitor valproate sodium. Compounds **29**–**31** exhibited antibacterial activities against *Bacillus subtilis*, with minimum inhibitory concentration (MIC) values of 5–10 μg/mL (Zhen et al., 2018).

**FIGURE 5:**
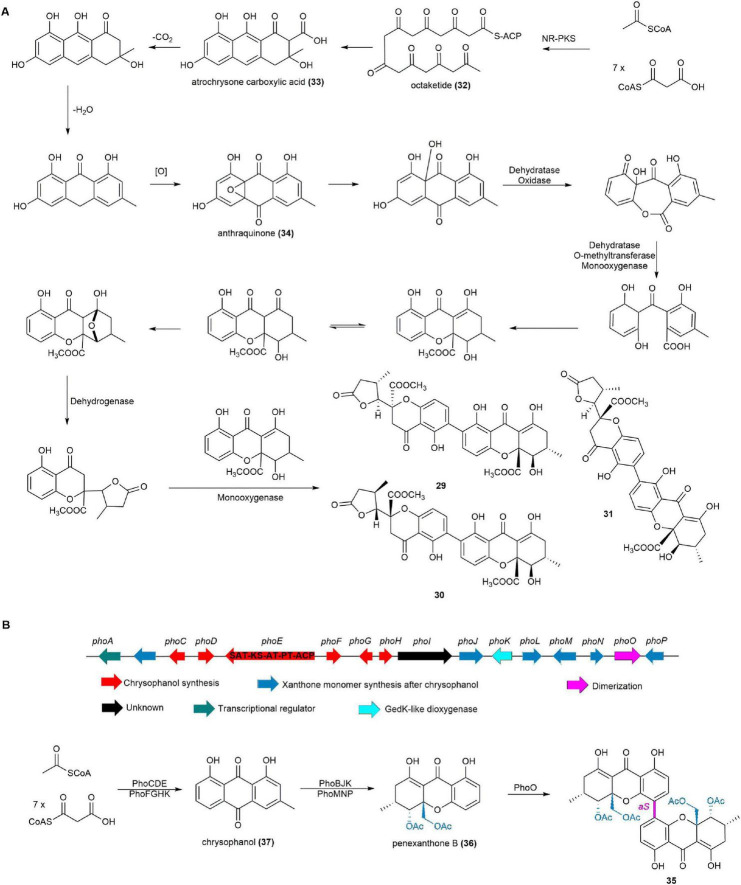
**(A)** The plausible biosynthetic pathway of chrysoxanthones. **(B)** The BGC and biosynthetic pathway of phomoxanthone A (**35**).

Following whole-genome sequencing of the fungus *P. chrysogenum* HLS111 and comparison with the known biosynthetic pathway of the tetrahydroxanthone dimer secalonic acid (Neubauer et al., 2016), a plausible biosynthetic pathway for chrysoxanthones was proposed. An iterative NR-PKS with KS-AT-PT-ACP architecture is responsible for synthesizing the octaketide (**32**). Atrochrysone carboxylic acid (**33**) is then released from the NR-PKS by a metallo-β-lactamase-type thioesterase (MβL-TE). This intermediate undergoes endogenous decarboxylation, dehydration, and oxidation to form anthraquinone (**34**). The final **29**–**31** are produced through successive dehydratase and oxygenase reactions (Figure 5A; Zhen et al., 2018).

#### 2.1.7 Phomoxanthone A

Phomoxanthone A (**35**), a homodimer of penexanthone B (**36**) formed through an unusual 4,4′-linkage, was isolated from various filamentous, including the ascidian-derived fungus *Diaporthe* sp. SYSU-MS4722 (Yuan et al., 2022b,Chen et al., 2022a). Compound **35** demonstrated superior cytotoxicity compared to cisplatin in both sensitive and resistant ovarian and bladder cancer cells. It induced mitochondrial depolarization, caspase activation, and apoptosis, specifically targeting the inner mitochondrial membrane without damaging plasma membranes. **35** also activated immune cells, potentially enhancing chemotherapy efficacy by overcoming resistance (Wang C. et al., 2019, Ronsberg et al., 2013, Frank et al., 2015). Additionally, **35** demonstrated antimicrobial activity against *Bacillus megaterium* and strong antifungal activity against the rice blast pathogen, *Pyricularia oryzae* (Elsässer et al., 2005).

The BGC, named *pho*, for **35** was definitively identified by completely deleting the *phoE* gene, a *pks* gene within the *pho* cluster potentially responsible for skeleton construction of **35**, in *Diaporthe* sp. SYSU-MS4722 using an advanced CRISPR/Cas9 system, resulting in the cessation of **35** production and confirming the pivotal role of the *pho* cluster in **35** biosynthesis. Heterologous expression of 14 biosynthetic genes in *A. oryzae* NSAR1 revealed that PhoCDEFGHK catalyzes the initial steps of **35** biosynthesis to give chrysophanol (**37**). Subsequently, PhoBJKLMNP process **37** to **36**. Feeding experiments indicated that PhoO, a cytochrome P450 enzyme, mediates the regioselective oxidative *para*-*para* coupling of **36** to yield **35** (Figure 5B; Yuan et al., 2022b).

#### 2.1.8 Amphichopyrones A and B

Amphichopyrones A (**38**) and B (**39**), α-pyrone derivatives isolated from *A. oryzae* NSAR1 constructs containing *amp* BGC from the ascidian-derived fungus *Amphichorda felina* SYSU-MS7908, have shown significant anti-inflammatory activity by inhibiting nitric oxide production in RAW264.7 cells, with IC_50_ values of 18.09 ± 4.83 μM and 7.18 ± 0.93 μM, respectively (Yuan et al., 2022a).

**FIGURE 6:**
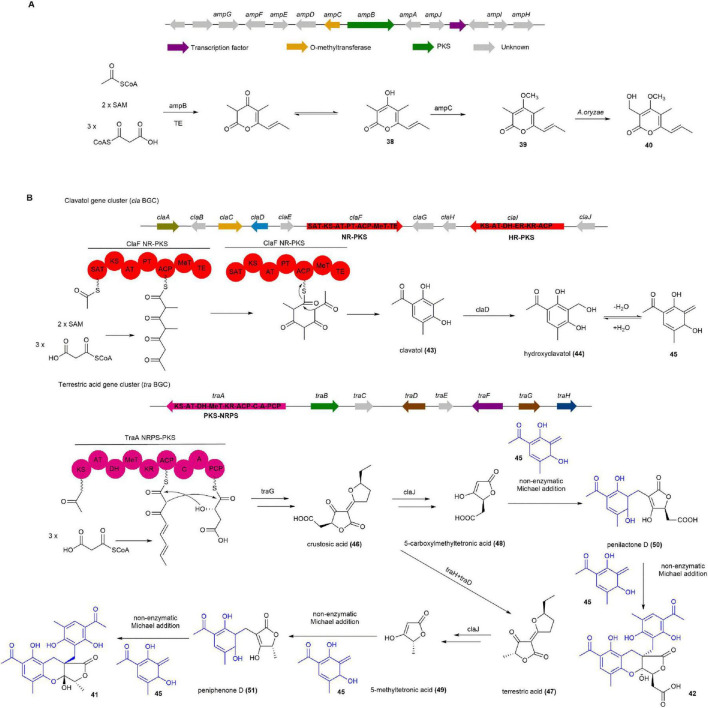
The BGCs and biosynthetic pathways of **(A)** amphichopyrone B (**39**) and **(B)** penilactone A (**41**) and B (**42**). SAT, starter unit:ACP transacylase; KS, ketosynthase; AT, acyltransferase; ACP, acyl carrier protein; PT, product template; DH, dehydratase; MeT, methyltransferase; ER, enoyl reductase; KR, ketoreductase; TE, thioesterase; A, adenylation; C, condensation; PCP, peptidyl carrier protein.

The *amp* cluster consists of 10 biosynthetic genes and shares similarities with the *sol* cluster, which is responsible for the biosynthesis of α-pyrone solanapyrone D (Kasahara et al., 2010). Introducing only the *ampB* gene into *A. oryzae* NSAR1 resulted in the production of **38**. When AmpC, a putative O-methyltransferase, was introduced into the AO-*ampB* construct, both **39** and udagawanone A (**40**) were produced. Adding the remaining eight genes, *ampADEFGHIJ*, to the AO-*ampBC* construct did not change the outcome, as **39** and **40** were still produced. These findings indicate that PKS AmyB is responsible for producing **38**, while AmpC catalyzes the methylation of **38** at the C-4 hydroxyl to form **39**. The subsequent hydroxylation of **39** to **40** is likely catalyzed by endogenous enzymes from the *A. oryzae* NSAR1 host (Figure 6A; Yuan et al., 2022a).

#### 2.1.9 Penilactones A and B

Penilactones A (**41**) and B (**42**), the highly oxygenated fungal polyketides about non-enzymatic Michael addition mediated the coupling process of polyketide–polyketide hybrids, were firstly isolated from an Antarctic deep-sea derived fungus *Penicillium crustosum* PRB-2 (Wu et al., 2012). Compound **41** showed NF-κB inhibitory activity with 40% inhibition rate at a concentration of 10 mM using transient transfection and reporter gene expression assays (Peng et al., 2012, Wu et al., 2012).

The biosynthetic pathway for **41** and **42** is proposed to originate from the hybridization of an *o*-quinone methide (**45**) unit and a γ-butyrolactone moiety through a 1,4-Michael addition, completing their carbon skeleton construction. Two separate BGCs, termed *cla* and *tra*, are responsible for this process, identified through gene deletion and heterologous expression in *A. nidulans*. After the deletion of *claF* or *traA*, the mutant strains completely abolished the production of **41** and **42**, suggesting that the associated BGC *cla* and *tra* are responsible for their biosynthesis. To determine the function of ClaF, *claF* was cloned into the expression vector pYH-wA-pyrG and expressed in *A. nidulans*. Clavatol (**43**) was successfully detected by LC-MS from the transformed *A. nidulans*. Furthermore, deletion of *claD* abolished the production of **41** and **42**, while **43** was clearly accumulated, indicating that the core NR-PKS ClaF in the *cla* BGC synthesizes **43**, which is subsequently oxidized by the non-heme Fe*^II^*/2-oxoglutarate-dependent oxygenase ClaD to form hydroxyclavatol (**44**). Subsequent gene knockout experiments on other genes within the *cla* and *tra* were carried out, leading to a comprehensive elucidation of the biosynthetic pathway for **41** and **42**. The subsequent biosynthetic pathway is as follows: **44** spontaneously dehydrates into the crucial intermediate **45**. In the *tra* BGC, the PKS-NRPS TraA and the trans-acting enoyl reductase (ER) TraG together form crustosic acid (**46**). The non-heme FeII/2-oxoglutarate-dependent oxygenase TraH then oxidatively decarboxylates **46** into dehydroterrestric acid, with its terminal double-bond reduced by the flavin-dependent oxidoreductase TraD to produce terrestric acid (**47**). Feeding experiments in a Δ*traA* mutant confirmed that **46** and **47** are intermediates that can be transformed into 5-carboxymethyl tetronic acid (**48**) and 5-methyltetronic acid (**49**), respectively. Notably, the enzyme(s) catalyzing the Michael addition were not identified. However, incubation of **48** with **44** at 25°C in water led to the formation of penilactone D (**50**) as the major product and **42** as the minor product. Similarly, incubation of **49** with **44** produced peniphenone D (**51**) as the major product and **41** as the minor product. Further incubation of **50** and **51** with **44** resulted in the formation of **41** and **42**. These findings indicate that the Michael addition in the biosynthesis of **41** and **42** occurs non-enzymatically and can happen spontaneously (Figure 6B; Dai et al., 2022, Fan et al., 2019, Fan et al., 2020).

#### 2.1.10 Alternapyrones G and H

Alternapyrones G (**52**) and H (**53**), α-pyrones with a 6-alkenyl chain, were isolated from a marine-derived strain of the fungus *Arthrinium arundinis*, and **52** not only suppressed M1 polarization in LPS-stimulated BV2 microglia but also stimulated dendrite regeneration and neuronal survival after Aβ treatment, suggesting its potential as a scaffold for Alzheimer’s disease drug discovery (Hu et al., 2024).

**FIGURE 7:**
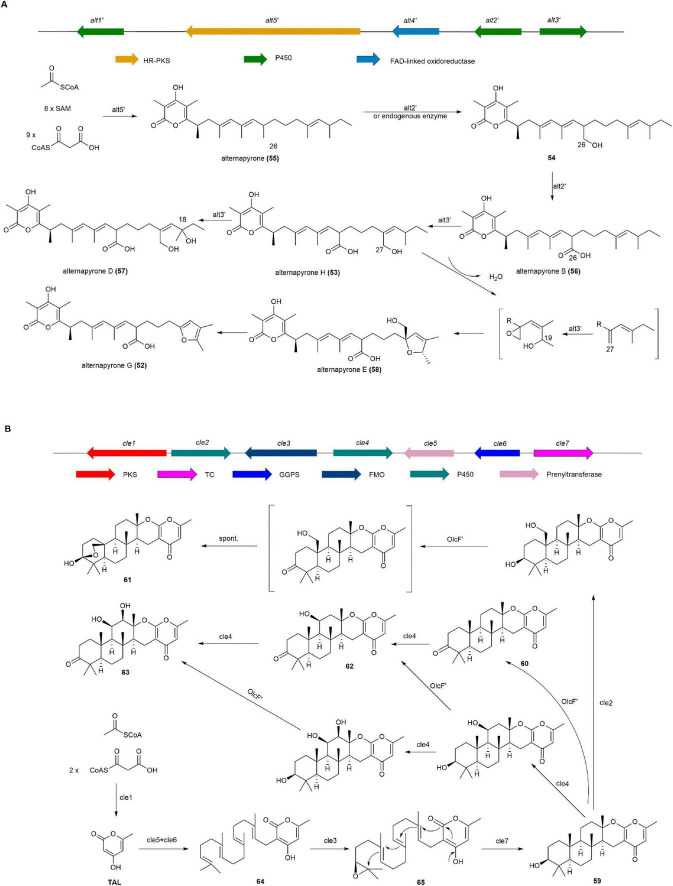
The BGCs and biosynthetic pathways of **(A)** alternapyrone G (**52**) and **(B)** chevalone E (**59**) and derivatives.

The BGC (*alt*′) for **52** and **53** was identified from *A. arundinis* ZSDS-F3 and validated by heterologous expression in *A. nidulans*. The *alt*′ BGC includes five open reading frames encoding a HR-PKS (alt5′), a flavin-linked oxidoreductase (alt4′), and three cytochrome P450 monooxygenases (alt3′, alt2′, and alt1′). The expression of HR-PKS alt5′ in *A. nidulans* led to the production of alternapyrone (**55**). Co-expression of alt5′ with alt1′ and alt4′ did not result in the formation of any new products, while co-expression of alt5′ with alt2′ and alt3′ led to the production of a set of products, including **53**, **54**, alternapyrone B (**56**), alternapyrone D (**57**), and alternapyrone E **(58**). Finally, the introduction of alt5′ along with the four alt genes (alt1′–4′) did not lead to the production of any new metabolites. Based on these results, the biosynthetic pathway of **52** and **53** are as follows: The HR-PKS Alt5′ synthesizes the polyketide chain from one acetyl-CoA, nine malonyl-CoA, and eight SAM molecules, followed by spontaneous lactonization to form **55**. The cytochrome P450 monooxygenase Alt2′ successive converts the methyl group at position 26 to a OH and carboxyl group, producing **54** and **56**. The cytochrome P450 monooxygenase Alt3′ then catalyzes successive hydroxylation, epoxidation, and oxidation steps to produce **52**, **53**, **57**, and **58** from **56** (Figure 7A; Hu et al., 2024).

### 2.2 Terpenes

#### 2.2.1 Chevalone E

Chevalone E (**59**), a class of meroterpenoids from the sponge fungus *Aspergillus milianensis* KUFA 0013, shows synergism with oxacillin against methicillin-resistant *Staphylococcus aureus* (MRSA) (Prompanya et al., 2014). It and its derivatives were discovered through heterologous expression of a cryptic gene cluster *cle* from *Aspergillus versicolor* 0312 in *A. oryzae* (Wang W.-G. et al., 2019). Additionally, chevalone analog, obtained *via* biocatalytic and chemical derivatization, such as chevalone F (**60**), N (**61**), O (**62**), and P (**63**), exhibit synergistic inhibition of MDA-MB-231 breast cancer cell viability when combined with doxorubicin (Xiao et al., 2022).

NR-PKS Cle1 was first expressed heterologously in *A. oryzae*, but no related products were detected. Co-expression of Cle1, Cle5, and Cle6 resulted in the production of **64**, indicating Cle1 generates TAL, while Cle5 and Cle6 are responsible for isopentenylation of side chains. Co-expression of Cle1, Cle5, Cle6, and FMO Cle3 produced the side chain epoxidation product **65**. Finally, **65** was converted to **59** by the cyclizing enzyme Cle7. Additionally, a series of **59** derivatives were obtained by expressing two P450 enzymes (Cle2 and Cle4) and a dehydrogenase OlcF′ from *A. felis* 0260 (Figure 7B; Wang W.-G. et al., 2019, Xiao et al., 2022).

#### 2.2.2 Ophiobolins

Ophiobolins are sesterterpenoids characterized by a 5-8-5 tricyclic skeleton, predominantly isolated from marine *Aspergillus* species, and exhibit notable cytotoxic properties (Zhang et al., 2012, Tian et al., 2017, Yan et al., 2022, Chai et al., 2016). Ophiobolin A (**66**) demonstrates efficacy against CLL and P388 cell lines, while ophiobolin O (**67**) inhibits MCF-7 proliferation and reverses MCF-7/ADR resistance to adriamycin (Bladt et al., 2013, Shen et al., 1999, Yang et al., 2012, Sun et al., 2013). **67** holds potential as a novel therapeutic agent and antagonist for multi-drug-resistant tumors, underscoring its significant clinical relevance for cancer chemotherapy (Sun et al., 2013, Yang et al., 2012). Additionally, 6-epi ophiobolin G (**68**) functions as an estrogen receptor down-regulator, offering potential for breast cancer treatment (Zhao et al., 2019). Ophiobolin G (**69**), ophiobolin H (**70**), ophiobolin K (**71**), 6-epi-ophiobolin K (**72**), **67**, and 6-epi-ophiobolin O (**73**) exhibit cytotoxicity against P388 cells, with IC_50_ values of 4.7, 9.3, 24.6, 105.7, 13.3 and 24.9 μM, respectively (Zhang et al., 2012). Notably, **66**, ophiobolin B (**74)**, ophiobolin C (**75)**, and **71** induce apoptosis in leukemia cells at nanomolar concentrations (Bladt et al., 2013).

**FIGURE 8:**
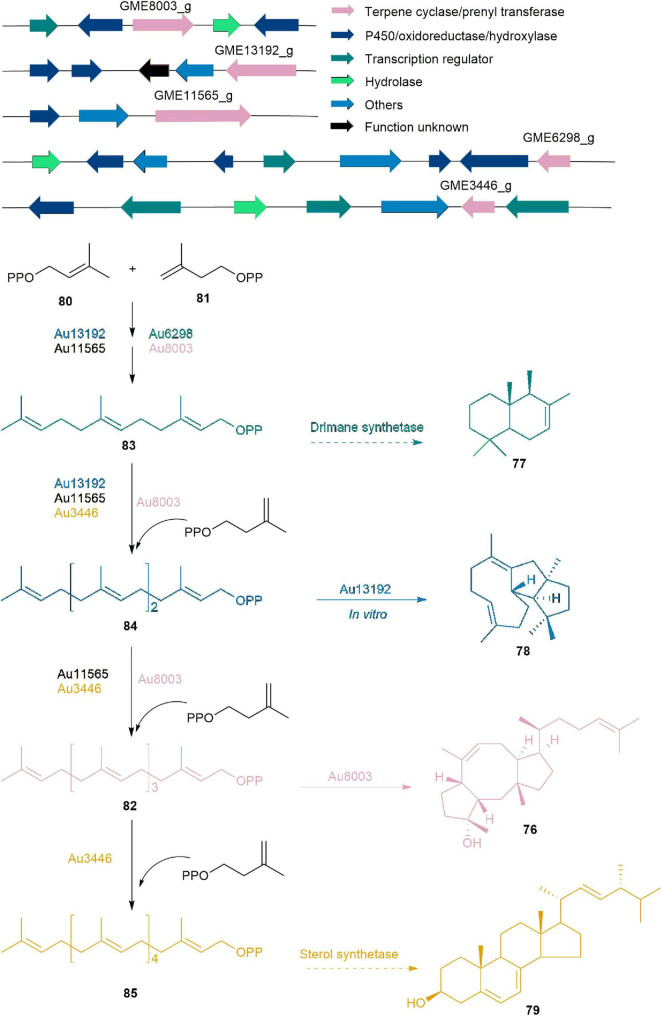
The BGCs and biosynthetic pathway of ophiobolin (**76**) and derivatives.

Five BGCs associated with ophiobolin (**76**) were identified through whole genome sequencing, gene inactivation, gene replacement, and *in vitro* enzyme catalysis experiments using endophytic fungus *Aspergillus ustus* 094102 derived from marine mangroves (Chai et al., 2016). They definitively established that these BGCs are responsible for producing natural products such as drimane (**77**), veridiene (**78**), **76**, and ergosterol (**79**) with carbon skeletons of C15, C20, C25, and C30, respectively. Among these clusters, Au8003 is pivotal in elongating chains from DMAPP (**80**) and IPP (**81**) to GFPP (**82**), and subsequently cyclizing **82** to yield **76**. The biosynthesis of **76** also involves complementary pathways, where Au6298, Au13192, and Au11565 catalyze the elongation of **80** and **81** to produce final products FPP (**83**), GGPP (**84**), and **82**, respectively. **83** could be used for **77** synthesis by drimane synthetase or for HexPP (**85**) synthesis by Au3446, which may then be used to synthesize **79**. Compound **84** produced by Au13192 serves as a crucial precursor not only for **76** but also for the production of **78** (Figure 8; Chai et al., 2016).

**FIGURE 9:**
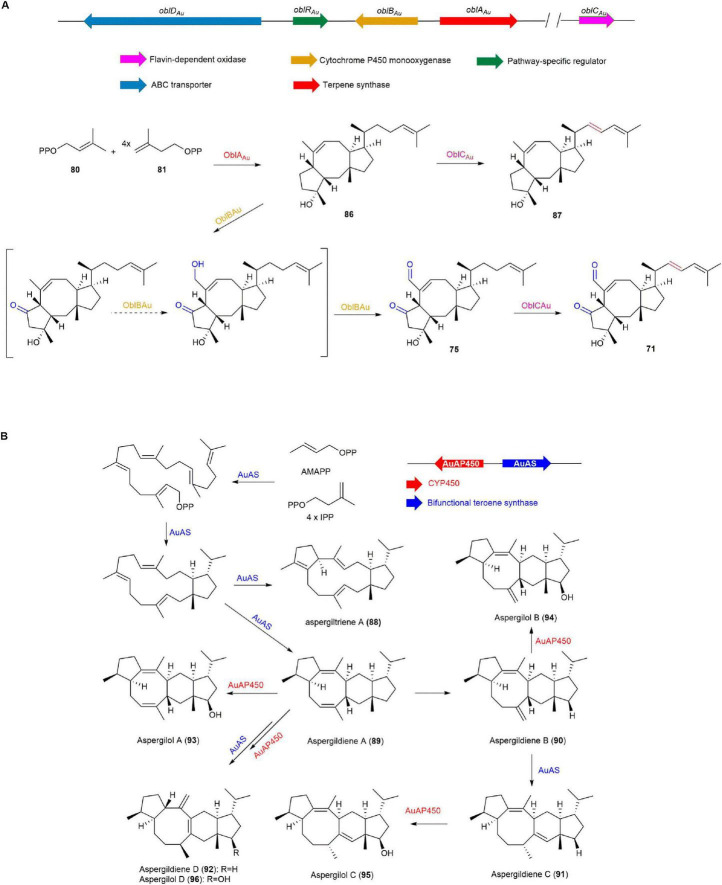
The BGCs and biosynthetic pathways of **(A)** ophiobolin K (**71**) and **(B)** aspergiltriene A (**88**) and derivatives.

In 2022, the biosynthetic pathway of **71** was elucidated through transcriptome analysis, gene knockout, heterologous expression, and precursor feeding experiments on *A. ustus* 094102 by the Hong group. The terpene synthase OblA_*Au*_ elongates and cyclizes **80** and **81** to form ophiobolin F (**86**), which is oxidized by the cytochrome P450 monooxygenase OblB_*Au*_ to **75**. The flavin-dependent oxidase OblC_*Au*_ catalyzes the conversion of **86** and **75** to 16,17-dehydro-ophiobolin F (**87**) and **71**, respectively. The transporter OblD_*Au*_ moves **71** and **75** between the cell wall and membrane, reducing their toxicity and preventing inhibition of host cells, thereby playing a detoxifying role (Figure 9A; Yan et al., 2022).

#### 2.2.3 Aspergildienes and aspergilols

In 2021, aspergildienes and aspergilols were discovered through genome mining of the marine-derived mangrove endophytic fungus *Aspergillus ustus* 094102 by the Hong group. Heterologous expression of AuAS, a bifunctional terpene synthase, in *A. oryzae* NSAR1, led to the discovery of five novel sesterterpenes, including a 5/12/5 tricyclic intermediate aspergiltriene A (**88**) and four 5/6/8/5 tetracyclic compounds aspergildiene A-D (**89**, **90**, **91**, **92**) with rare stereochemistry. Coexpression with the upstream cytochrome P450 monooxygenase (AuAP450) led to the discovery of four new corresponding sesterterpene alcohols aspergilol A-D (**93**, **94**, **95**, **96**). Among these, **93** was found to exhibit cytotoxicity against MCF-7, MDA-MB-231, and HepG2 cancer cells (IC_50_ 21.20-48.76 μM), while **94** demonstrated cytotoxic effects specifically on MCF-7 cells (IC_50_ 27.41 μM) (Figure 9B; Guo et al., 2021).

#### 2.2.4 Spiromaterpenes

**FIGURE 10:**
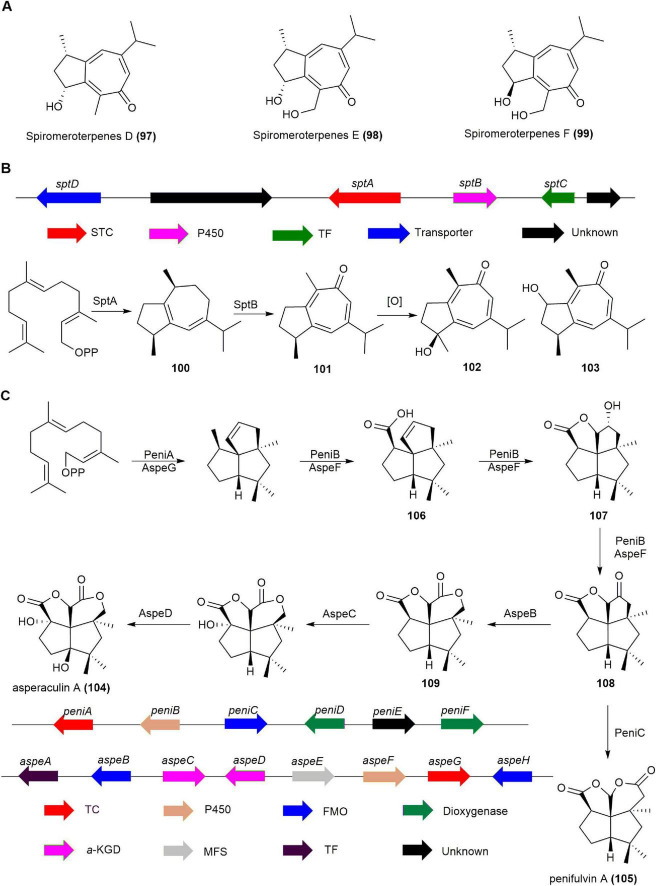
**(A)** The structures of spiromeroterpenes D-F (**97**, **98**, **99**). **(B)** The BGC and biosynthetic pathway of spiromaterpenes. **(C)** The BGCs and biosynthetic pathway of penifulvin A (**105**).

Spiromaterpenes, guaiane-type sesquiterpenes, emerged from the activation of a terpene-related BGC following the epigenetic manipulation of a deep-sea sediment-derived *Spiromastix* sp. fungus using suberoylanilide hydroxamic acid (SAHA). Spiromeroterpenes D-F (**97**, **98**, **99**) effectively inhibited NO production in LPS-induced BV2 microglial cells, with preliminary structure-activity relationship indicating that the 2(*R*),11-diol unit enhances their efficacy (Figure 10A). Notably, **98** prevented the LPS-induced translocation of NF-κB from the cytosol to the nucleus, and significantly reduced pro-inflammatory cytokines IL-1β, IL-6, and TNF-α, as well as iNOS and COX-2 at both the protein and mRNA levels in BV2 cells. These results highlight **98**′s potential as a promising agent for further development in combating neuroinflammation (Guo et al., 2021).

The biosynthetic pathway of spiromeroterpenes in *Spiromastix* sp. was elucidated by heterologous expression, biochemical characterization, and incubation experiments. Co-expression of the sesquiterpene cyclase SptA, a homologous protein of the known fungal guaiane-type sesquiterpene cyclase FfSTC5 (Burkhardt et al., 2016), and cytochrome P450 SptB in *A. nidulans* LO8030 successfully produced spiromeroterpene A (**101**) and its derivatives **102** and **103**. Subsequently, SptA was expressed and purified in *Escherichia coli*, then incubated with FPP and Mg^2+^, yielding compound **100**. By introducing the P450 enzyme SptB separately into *A. nidulans* LO8030 and using **100** as a substrate, the target product **101** and its derivatives **102** and **103** were also obtained. These findings suggest that SptA catalyzes the production of guaia-1(5),6-diene, while cytochrome P450 SptB is responsible for the formation of the tropone ring (Figure 10B; Liu et al., 2022).

#### 2.2.5 Asperaculin A

Asperaculin A (**104**), a sesquiterpenoid with a unique [5,5,5,6] dioxafenestrane ring system, was isolated from the marine fungus *Aspergillus aculeatus* CRI323-04. It closely resembles penifulvin A (**105**) from the terrestrial fungus *Penicillium griseofulvum* NRRL35584 but is distinguished by a transposed γ-lactone ring and an additional hydroxyl group at C9 (Ingavat et al., 2011, Das and Chakraborty, 2016).

The BGC known as *aspe* in *Aspergillus aculeatus* CRI323-04, which is homologous to the BGC *peni* for **105** with a similar dioxa [5.5.5.6] fenestrane core, was confirmed to be responsible for **104** biosynthesis through heterologous expression in *A. nidulans*. Heterologous reconstruction of *aspe* and *peni* clusters in *A. nidulans* showed that the sesquiterpene synthases (PeniA and AspeG) and cytochrome P450 enzymes (PeniB and AspeF) perform identical functions, producing intermediates **106**, **107**, and **108**. Co-expression of *aspeGFB* in *A. nidulans* resulted in the generation of oxidation product **109**, while constructs harboring *aspeGF* + *peniC* produced **105**. This indicates that PeniC and AspeB selectively undergo Baeyer–Villiger oxidation at different positions of the same substrate **108** to generate distinct esterification products, compounds **105** and **109**. The final product, compound **104**, is formed through the action of two dioxygenases, AspeCD (Figure 10C; Wei et al., 2021b,Zeng et al., 2019, George et al., 2021).

#### 2.2.6 Talaronoids

Talaronoids, fusicoccane diterpenoids with a unique tricyclic 5/8/6 ring system, were discovered from the marine-derived fungus *Aspergillus flavipes* CNL-338 (Zhang et al., 2022). Talaronoids A–D (**110**, **111**, **112**, **113**) showed butyrylcholinesterase (BChE) inhibitory activity with IC_50_ values of 14.71 ± 1.07, 26.47 ± 0.35, 31.51 ± 0.28, and 11.37 ± 0.85 μM, respectively (Zhang et al., 2020).

**FIGURE 11:**
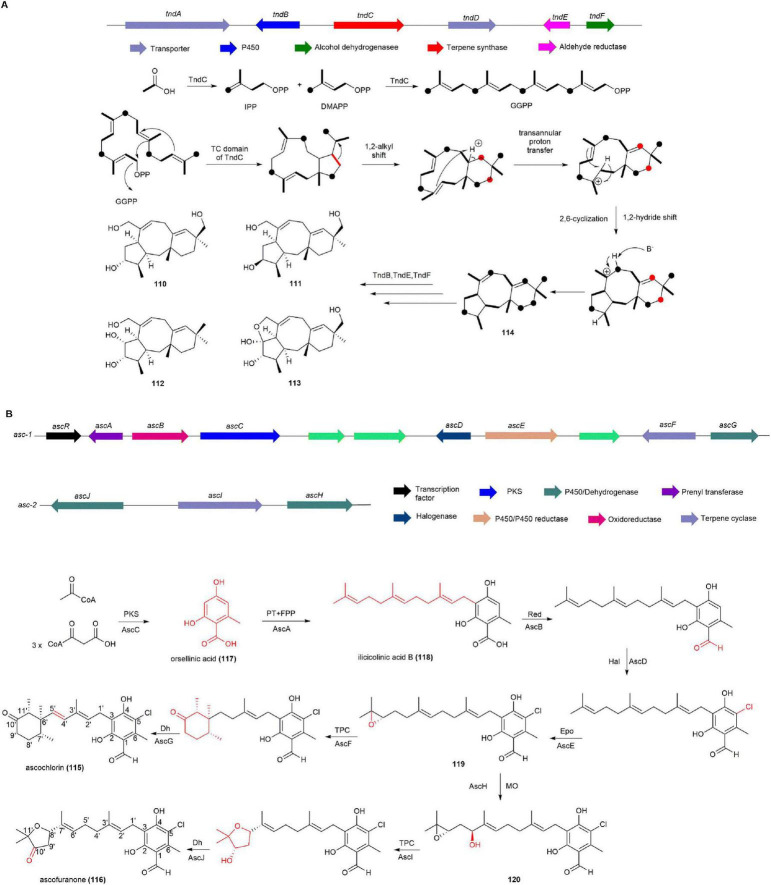
The BGCs and biosynthetic pathways of **(A)** talaronoids and **(B)** ascochlorin (**115**) and ascofuranone (**116**).

After sequencing the whole genome of *A. flavipes* CNL-338, the BGC known as *tnd*, responsible for talaronoid production, was confirmed through heterologous expression. The *tndC* gene, encoding a protein homologous to the known diterpene synthase PaFS (Toyomasu et al., 2007) and the sesterterpene synthase AcOS (Chiba et al., 2013), was expressed in *Saccharomyces cerevisiae*, leading to the detection of talarodiene (**114**). Stable isotope tracer experiments further demonstrated the conversion of geranylgeranyl diphosphate to **114**, suggesting that TndC is a novel bifunctional diterpene synthase. Finally, a cytochrome P450 enzyme (TndB), an aldehyde reductase (TndE), and an alcohol dehydrogenase (TndF) were proposed to collectively catalyze the conversion of **114** into compounds **110**, **111**, **112**, **113** (Figure 11A; Zhang et al., 2022).

### 2.3 Meroterpenoids

#### 2.3.1 Ascochlorin and ascofuranone

Ascochlorin (**115**) is a meroterpenoid with 5-chloroorcylaldehyde substituted at C-3 by a cyclized sesquiterpene side chain, extractable from marine-derived fungus *Acremonium Sclerotigenum* (Luo et al., 2021) and *Stilbella fimetaria* (Subko et al., 2021). **115** and its derivatives exhibit a wide range of physiological activities, including antibacterial, antitumor, antiviral, hypolipidemic, antihypertensive, and anti-inflammatory effects, as well as improving type I and II diabetes by reducing serum cholesterol and triglyceride levels (Kawaguchi et al., 2013, Luo et al., 2021, Subko et al., 2021, Lee et al., 2016, Magae et al., 1982, Seephonkai et al., 2004, Tamura et al., 1968, Takatsuki et al., 1969, Magae et al., 1988, Sasaki et al., 1973). Additionally, ascofuranone (**116**) shows promise as a candidate for treating *African trypanosomiasis* (Yabu et al., 2003, Shiba et al., 2013).

The BGCs of **115** and **116** have been identified through transcriptome analysis, gene knockout, and heterologous expression in the fungus *Acremonium egyptiacum*. The production of **115** and **116** in *A. egyptiacum* varied depending on the culture medium, with 0.96 mg of **116** produced in F1 medium and 399 mg of **116** in AF medium. After isolating poly(A)-selected RNAs from mycelia grown in both F1 and AF media and conducting transcriptome analysis, it was found that the expression of genes (*ascABCDEFGR*) in the *asc-1* cluster and genes (*ascHIJ*) in the *asc-2* cluster were more strongly induced in AF medium than in F1 medium. This suggests that *asc-1* and *asc-2* clusters are responsible for the biosynthesis of **115** and **116**. Further, heterologous expression in *A. oryzae* and targeted gene knockouts in *asc-1* and *asc-2* were performed to fully elucidate the biosynthetic pathways of **115** and **116**. *Asc-1* comprises eight genes, including NR-PKS AscC responsible for producing the precursor orsellinic acid (**117**). AscA, an isopentenyl transferase, catalyzes the formation of ilicicolinic acid B (**118**) from farnesyl pyrophosphate (FPP) and **117**, which undergoes subsequent reduction by AscB, chlorination by AscD, and epoxidation by AscE to form compound **119**, which is then converted to **116** by the terpenoid cyclase AscF and the oxidase AscG. In addition, **119**, recognized by the P450 enzyme AscH from *asc-2*, undergoes hydroxylation at its isopentenyl group, leading to the formation of **120**. Subsequently, **120** undergoes cyclization catalyzed by the terpenoid cyclase *Asc*I, followed by oxidation by AscJ, resulting in the production of **116** (Figure 11B; Araki et al., 2019).

#### 2.3.2 Chrodrimanins, verruculides, and talaromyides

Chrodrimanins, verruculides, and talaromyides, polycyclic meroterpenoids with a seco-drimane unit and an isocoumarin core, have been isolated from marine-derived fungi *Talaromyces* sp. CX11 (Cao et al., 2019), *Talaromyces purpureogenus* (Cao et al., 2020), *Penicillium* sp. SCS-KFD09 associated with the marine worm *Sipunculus nudus* (Kong et al., 2017), and ascidian-derived *Penicillium verruculosum* TPU1311 (Yamazaki et al., 2015). Talaromyolide D (**121**) exhibits potent antiviral activity against pseudorabies virus (PRV) with a CC_50_ of 3.35 μM (Cao et al., 2019), while talaromyolide I (**122**) and K (**123**) show dose-dependent inhibition of PRV, with **123** demonstrating the most significant effects at 50 mg/mL (Cao et al., 2020). Chrodrimanin O (**124**), R (**125**), S (**126**), verruculide A (**127**), and chrodrimanin A (**128**), B (**129**), and H (**130**) exhibit protein tyrosine phosphatase 1B (PTP1B) inhibitory activity, with IC_50_ values ranging from 71.6 to 8.4 μM, suggesting potential for development as drugs targeting type 2 diabetes or obesity (Kong et al., 2017, Yamazaki et al., 2015).

**FIGURE 12:**
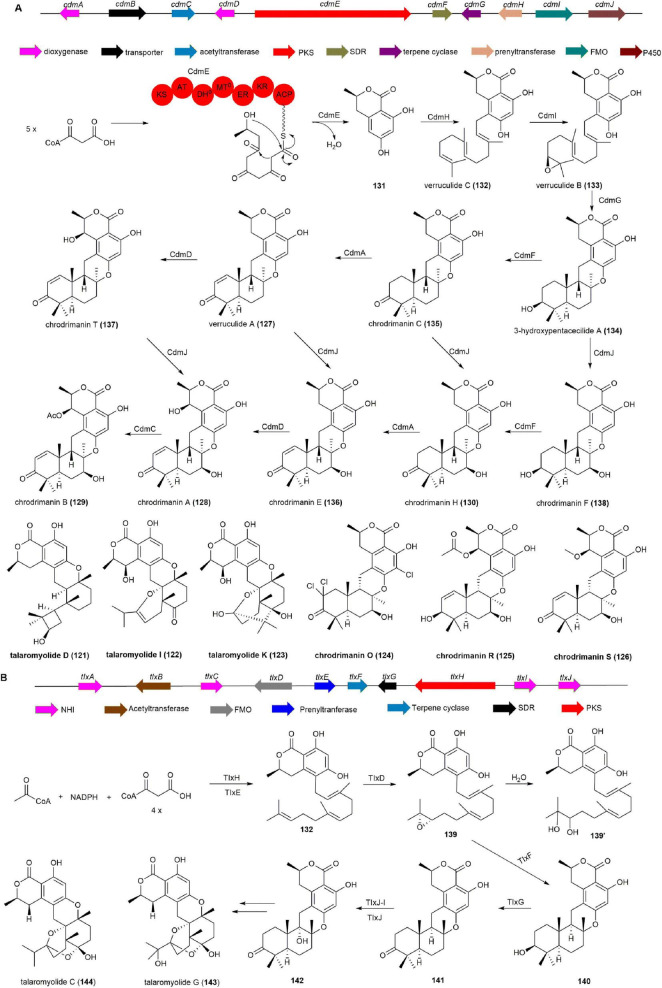
**(A)** The BGC, biosynthetic pathway and structures of chrodrimanins, verruculides and talaromyides. **(B)** The BGC and biosynthetic pathway of talaromyolide G (**143**) and C (**144**).

The BGC responsible for **129**, designated as the *cdm* cluster, underwent characterization through whole genome sequencing, heterologous reconstitution in *A. oryzae*, and *in vitro* enzyme reactions. Initially, the PKS CdmE, serving as the 6-hydroxymellein synthase, was expressed in *A. oryzae*, resulting in the production of **131**. Co-expression of *cdmE* with the prenyltransferase gene *cdmH* yielded the hydrophobic metabolite verruculide C (**132**). Subsequent incorporation of the FMO gene *cdmI* yielded verruculide B (**133**), while introduction of the terpene cyclase gene *cdmG* generated the pentacyclic molecule 3-hydroxypentacecilide A (**134**). Integration of *cdmF* into *A. oryzae* producing compound **134** led to the production of chrodrimanin C (**135**), confirming CdmF as a 3-hydroxy dehydrogenase. Additionally, the Fe(II)/α-ketoglutarate (αKG)-dependent dioxygenase CdmA exhibited dehydrogenation activity between C-1 and C-2 in **135** and **130**, resulting in the formation of **127** and chrodrimanin E (**136**). Furthermore, CdmD, another Fe(II)/αKG-dependent dioxygenase, catalyzed β-hydroxylation at C-7′ to produce chrodrimanin T (**137**) and **128**. The cytochrome P450 monooxygenase CdmJ accepted compounds **127**, **134**, **135** and **137** as substrates, acting as a C-7 β-hydroxylase to produce chrodrimanin F (**138**), **130**, **136** and **128**, respectively. Finally, the acetyltransferase CdmC converted compound **128** into the final product **129** (Figure 12A; Bai et al., 2018).

The BGC responsible for the production of talaromyolides, a unique group of 6/6/6/6/6/6 hexacyclic meroterpenoids, was identified in the marine fungus *T. purpureogenus* and designated as the *tlx* cluster. As expected, compound **131**, a precursor molecule of talaromyolides, was produced in *A. oryzae* harboring the PKS TlxH, which shares 66% homology with CdmE (Bai et al., 2018). Coexpression of *tlxH* and *tlxE* led to the production of **132**. Subsequent introduction of *tlxD* in *A. oryzae* harboring *tlxHE* resulted in the production of dihydroxyfarnesyl-9 (**139′**) presumably derived from **139** by epoxide opening through attack of water. The transformant expressing *tlxHEDF* yielded **140**, Further introduction of *tlxG* in the *A. oryzae* harboring *tlxHEDF* resulted in the generation of **141**. Ultimately, the heterodimer of non-heme iron (NHI) enzyme TlxJ catalyzed the hydroxylation of **141** at C-9α to produce **142**, and co-incubation with TlxI efficiently yielded the target products talaromyolide G (**143**) and C (**144**) (Figure 12B; Li et al., 2021).

### 2.4 Non-ribosomal peptides

#### 2.4.1 Gliotoxin

Gliotoxin (**145**), featuring a diketopiperazine core with a disulfide bridge, is isolated from various fungal species, including marine fungus *Neosartorya pseudofischeri* found in the inner tissue of the starfish *Acanthaster planci* (Liang et al., 2014, Scharf et al., 2016). Compound **145** exhibits a diverse range of biological activities, such as antimicrobial, antifungal, antiviral, and immunomodulating properties (Scharf et al., 2016, Waring and Beaver, 1996). **145** and dithiol gliotoxin (**146**) show significant inhibitory activity against Gram-positive *Staphylococcus aureus* (ATCC29213) and methicillin-resistant *Staphylococcus aureus* (R3708), as well as Gram-negative *Escherichia coli* (ATCC25922), with MIC values ranging from 1.52 to 97.56 μM, and notably exhibit potent inhibition against *Staphylococcus aureus* R3708 with MIC values of 1.53 and 1.52 μM, respectively (Liang et al., 2014). Structure-activity relationship analysis suggests that the disulfide bridge or its reduced form is essential for antibacterial activity, which is influenced by modifications on the six-membered ring with two conjugated double bonds, where a hydroxyl group at C-6 enhances activity compared to an acetyl group. The α-methylene ketone group is also crucial for antibacterial activity (Liang et al., 2014). Furthermore, **145** and **146** also demonstrate excellent cytotoxic activity against the human embryonic kidney (HEK) 293 cell line and human colon cancer cell lines, HCT-116 and RKO, with IC_50_ values of 0.41 and 1.58 μM, respectively (Liang et al., 2014, Watts et al., 2010, Waring et al., 1995, Waring and Beaver, 1996).

**FIGURE 13:**
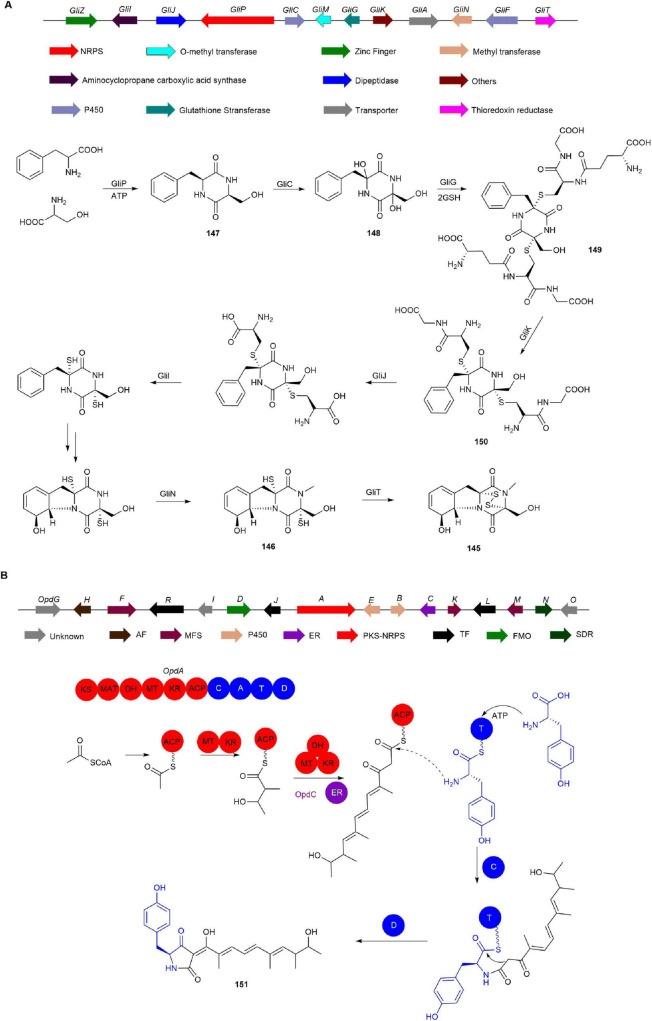
The BGCs and biosynthetic pathways of **(A)** gliotoxin (**145**) and **(B)** oxopyrrolidine A (**151**).

The BGC of **145**, known as *gli* and consisting of 13 genes, was identified by the Howlett group through whole genome sequencing and bioinformatics analysis of *Aspergillus fumigatus* (Gardiner and Howlett, 2005). Within this cluster, GliZ, a transcription factor, upregulates gliotoxin biosynthesis (Bok et al., 2006). Furthermore, the NRPS GliP catalyzes the production of the precursor **147** (Balibar and Walsh, 2006), which is subsequently hydroxylated by GliC to form **148** (Chang et al., 2013). Additionally, GliG, a glutathione *S*-transferase, catalyzes the formation of **149** from **148** and two molecules of glutathione, providing the sulfur source for **145** (Davis et al., 2011, Scharf et al., 2011). Then, glutamic acid transferase GliK removes glutamyl to generate **150**, which is further modified by GliI and methyltransferase GliN to produce **146** (Gallagher et al., 2012, Scharf et al., 2013, Scharf et al., 2012). Finally, the oxidoreductase GliT catalyzes the formation of disulfide bridges, yielding the final product **145** (Figure 13A; Scharf et al., 2014).

#### 2.4.2 Oxopyrrolidines

Tetramic acid derivatives, featuring a pyrrolidine-2,4-dione moiety, are crucial in medicinal chemistry and biochemistry due to their antibiotic (Segeth et al., 2003), antifungal (Sata et al., 1999), and cytotoxic activities (Holzapfel, 1968). Oxopyrrolidines, a type of tetramic acid derivative, have been isolated from the marine-derived fungus *Penicillium oxalicum* MEFC104 (Li et al., 2022).

Bioinformatic analysis of the aspyridone gene cluster, which has a structure similar to oxopyrrolidine A (**151**), identified the candidate *opd* gene cluster responsible for **151** production in *P. oxalicum* MEFC104 (Bergmann et al., 2007). The *opd* cluster was further confirmed by inactivating the PKS-NRPS gene *opdA*, resulting in a mutant that lost the ability to produce **151**. Further analysis of the 16 genes within the *opd* BGC identified OpdJ, OpdL, and OpdR as transcription factors. **151** was absent in the Δ*opdJ* mutant, while mutants without *opdL* or *opdR* showed no significant changes, indicating that OpdJ is the cluster-specific transcription factor regulating the *opd* cluster. Deletion of the MFS transporter genes *opdF*, *opdK*, and *opdM* did not affect **151** biosynthesis, suggesting these transporters are not involved in **151** production. Of the remaining eight genes (*opdBCDEGNOI*), only the Δ*opdC* mutant completely lost the ability to produce **151**, with no accumulation of intermediates. This indicates that OpdC acts as a trans-acting ER essential for the reduction step in the polyketide assembly process. Thus, the biosynthesis of **151** primarily relies on the actions of OpdA and OpdC (Figure 13B; Li et al., 2022).

#### 2.4.3 Psychrophilins

Psychrophilins, featuring a rare amide linkage between the carboxylic acid in anthranilic acid (ATA) and the nitrogen from an indole moiety, were isolated from the marine-derived fungus *Aspergillus versicolor* ZLN-60 and marine algae-derived fungi of the genus *Aspergillus* (Ebada et al., 2014, Peng et al., 2014). Psychrophilin G (**152**) exhibits potent lipid-lowering effects in HepG2 hepatocarcinoma cells (IC_50_ = 10 μg/mL) (Peng et al., 2014). Psychrophilin E (**153**) shows strong anti-proliferative activity against the HCT116 (colon) cell line (IC_50_ = 28.5 μg/mL) with high selectivity and demonstrates more potent cytotoxic activity than cisplatin, a clinically used chemotherapeutic agent (IC_50_ = 33.4 μg/mL) (Ebada et al., 2014, Ngen et al., 2016).

**FIGURE 14:**
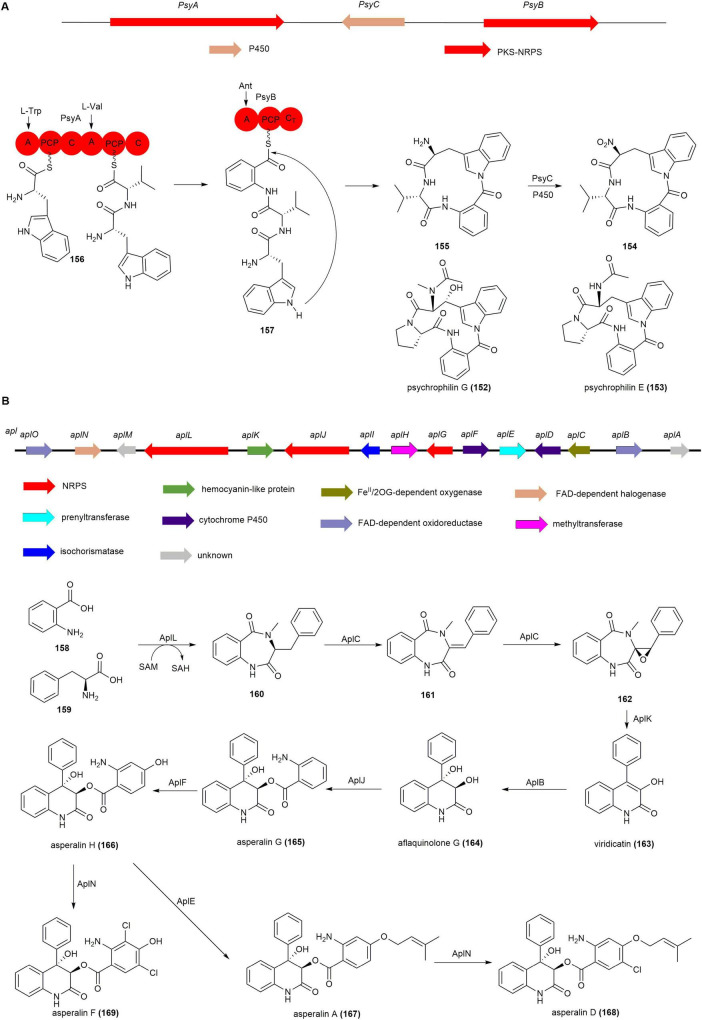
The BGCs and biosynthetic pathways of **(A)** psychrophilins and **(B)** asperalins.

Sequencing the genome of the psychrophilin B (**154**) producing fungus *Penicillium rivulum* revealed two candidate BGCs encoded in scaffold 46 and scaffold 182 as likely involved in **154** biosynthesis (Dalsgaard et al., 2004, Zhao et al., 2016). Gene knockout of scaffold 46 using homologous recombination resulted in the complete abolishment of **154** production, confirming scaffold 46 as the responsible gene cluster, named *psy*. The *psy* cluster contains two independent NRPS coding genes *psyA* and *psyB*. Single-gene knockouts of *psyA* and *psyB* resulted in the complete loss of **154** production, with no related intermediates detected. However, in the P450 deletion strain Δ*psyC*, psychrophilin I (**155**) was detected. Feeding **155** to the Δ*psyA* and Δ*psyB* strains led to the detection of the target **154**, suggesting **155** as the penultimate intermediate in **154** biosynthesis. Based on genetic inactivation and chemical complementation studies, the proposed biosynthetic pathway for **154** is as follows: the dimodular NRPS PsyA incorporates L-Trp and L-Val to yield the L-Trp–L-Val dipeptidyl thioester (**156)**. The monomodular NRPS PsyB activates Ant, which is then condensed with **156** by the terminal C domain in PsyA to yield the tripeptidyl thioester (**157**). The CT domain of PsyB utilizes the indole nitrogen in a nucleophilic attack of the thioester to release **155**, which is then catalyzed by P450 PsyC to form **154** (Figure 14A; Zhao et al., 2016).

#### 2.4.4 Asperalins

Asperalins, viridicatin-type quinolone alkaloids, are significant natural products with various biological activities, including insecticidal (Uchida et al., 2006), antibacterial (Hu et al., 2023), antifungal (Mousa et al., 2015), antitumor (Larsen et al., 2002, He et al., 2005), and antiviral properties (Chen et al., 2014). Recently, Gao group isolated novel asperalins from the seagrass-derived fungus *Aspergillus alabamensis* SYSU-6778, which exhibit moderate to potent inhibitory effects against fish pathogenic bacteria, such as *Edwardsiella ictaluri*, *Streptococcus iniae*, and *Streptococcus parauberis* (Hu et al., 2023).

The asperalins BGC, named as *apl*, from *A. alabamensis* SYSU-6778 was confirmed *via* heterologous expression in *A. oryzae* NSAR1, incorporating aspects of viridicatin-type quinolone alkaloid biosynthesis (Kishimoto et al., 2018, Zou et al., 2017, Zou et al., 2015). Heterologous expression of AplLCK in *A. oryzae* NSAR1resulted in the detection of **160**, **161**, **162** and viridicatin (**163**), indicating that the pathway of asperalins initiates with the dual-module NRPS aplL. AplL catalyzes the condensation of o-aminobenzoic acid (**158**) and L-phenylalanine (**159**) to form **160**, which is then converted by the dioxygenase aplC into **161** and subsequently epoxidized to form **162**. A zinc-dependent protein aplK facilitates the ring contraction of **162**, producing **163** through the elimination of methyl isocyanate. Feeding **163** into AO-AplB constructs results in aflaquinolone G (**164**), generated by hydroxylation *via* the FAD-dependent monooxygenase aplB. The NRPS aplJ transforms **164** into asperalin G (**165**), which is subsequently processed by the P450 enzyme aplF into asperalin H (**166**). Compound **166** undergoes *O*-prenylation by aplE to produce asperalin A (**167**), while chlorase aplN converts both **167** into asperalin D (**168**) and **166** into asperalin F (**169**) (Figure 14B; Zeng et al., 2024).

### 2.5 Alkaloids

#### 2.5.1 Isoindolinones

Isoindolinones, isolated from the marine fungus *Stachybotrys longispora* FG216, are known for their potent plasminogen-activating properties (Shinohara et al., 1996, Hu et al., 2001, Hasegawa et al., 2010, Koide et al., 2012, Yin et al., 2017). Isoindolinones exhibit strong fibrinolytic effects and have shown promising results in treating thrombotic strokes in primates, enhancing thrombolysis and minimizing hemorrhagic activity (Hasegawa et al., 2010, Hu et al., 2012). Consequently, isoindolinones hold significant potential for the development of cardiovascular drugs (Sawada et al., 2014, Yan et al., 2015).

**FIGURE 15:**
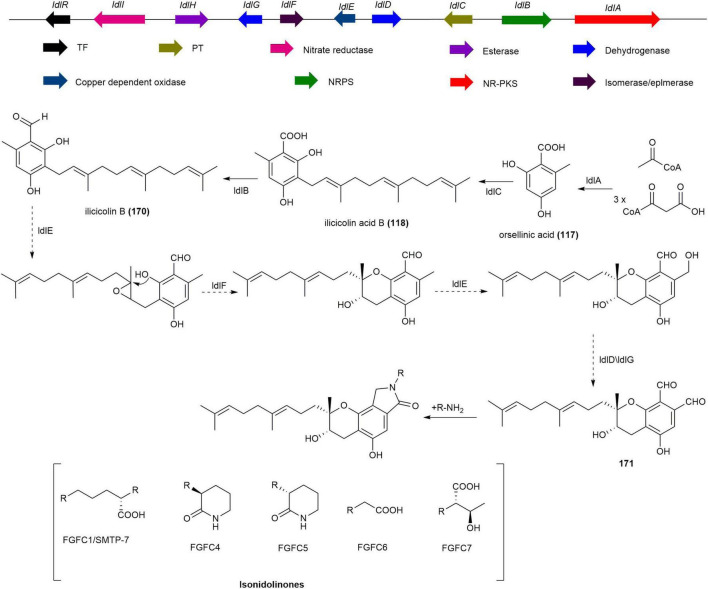
The BGC and biosynthetic pathway of isoindolinones.

Ilicicolin B (**170**), synthesized by NR-PKS StbA, UbiA-like prenyltransferase StbC, and NRPS-like enzyme StbB in *Stachybotrys bisbyi* PYH05-7, is the precursor of all isoindolinone derivatives (Nishimura et al., 2012, Li et al., 2016). Based on these core genes (*stbABC*), the BGC of isoindolinones was identified in *S. longispora* FG216 through genome mining (Yin et al., 2017). The biosynthetic pathway of isoindolinones, as deduced from bioinformatics analysis, starts with the synthesis of orsellinic acid (**117**) by NR-PKS IdlA, followed by the transfer of farnesyl pyrophosphate (FPP) by PT IdlC to form ilicicolin acid (**118**), which is then converted by NRPS IdlB into **170**. Finally, through epoxidation, cyclization, and oxidation steps, the phthalic aldehyde precursor (**171**) is formed, which combines with ammonium ions or amino compounds to produce various isoindolinones (Figure 15; Yin et al., 2017).

## 3 Conclusion

MFNPs represent a significant source of pharmaceuticals, exhibiting remarkable bioactivity and therapeutic potential. With the rapid advancement of genomic sequencing technologies, genome mining has emerged as a crucial strategy for discovering new MFNPs. The BGCs was primarily identified by comparative transcriptome analysis (ophiobolins, ascochlorin and ascofuranone) and bioinformatic analysis of the sequenced genome of producing strains. Heterologous expression in *Saccharomyces cerevisiae*, *Aspergillus nidulans*, and *Aspergillus oryzae*, along with gene knockout techniques in producing strains, are essential for unlocking these dormant biosynthetic pathways. The majority of these MFNPs discussed in this review are derived from the genera *Penicillium* (griseofulvin, sorbicillinoids, monodictyphenone, chrysoxanthones, penilactones A, penilactones B, chrodrimanins, verruculides, talaromyides, penifulvin A and oxopyrrolidines) and *Aspergillus* (epicospirocins, chevalone E, ophiobolins, aspergildienes, aspergilols, asperaculin A, talaronoids, psychrophilins and asperalins). The pharmacological activities of these MFNPs are prominently featured in anti-inflammatory activities (flavoglaucin, dihydroauroglaucin, isodihydroauroglaucin, sorbicillinoids, amphichopyrone A, amphichopyrone B, penilactones A, ascochlorin, spiromeroterpenes D-F), cytotoxic activities (flavoglaucin, aspermicrones B, phomoxanthone A, oxopyrrolidines, psychrophilin G, psychrophilin E, ophiobolins and aspergilols), and antimicrobial activities (griseofulvin, monodictyphenone, aspermicrone B, aspermicrone C, chrysoxanthones A-C, phomoxanthone A, chevalone E, ascochlorin, gliotoxin, oxopyrrolidines). The research efforts outlined in this review offer valuable perspectives for future gene-guided mining and analysis of biosynthetic pathways in MFNPs.

## 4 Discussion and outlook

MFNPs represent a rich source of structurally diverse bioactive compounds with significant therapeutic potential. Notable examples, such as ziconotide (Prialt), trabectedin (Yondelis), and lurbinectedin (Zepzelca), are marine-derived drugs that continue to offer substantial benefits to human health. However, the discovery of novel MFNPs has been hindered by challenges in current discovery technologies, cultivation methods, and screening models, which often lack integration with genomic approaches (Atanasov et al., 2021). Consequently, MFNPs remain underexplored relative to their synthetic counterparts, limiting their full potential in drug development. Recent advances in genome mining, including gene editing, gene synthesis, and heterologous expression systems, have revolutionized the discovery of marine fungal natural products (MFNPs) by enabling the identification of previously cryptic BGCs (Costantini, 2020, Wei et al., 2021a). The increasing availability of sequenced marine fungal genomes has uncovered a wealth of untapped BGCs, and when coupled with advanced bioinformatics tools, these resources significantly enhance the efficiency of bioactive MFNPs identification. Moreover, the elucidation of biosynthetic pathways lays the groundwork for metabolic engineering strategies that can optimize the production of these compounds, addressing the low natural yields often encountered in MFNP discovery.

Despite these advancements, several challenges persist in genome mining: (1) Some BGCs remain silent, even with multiple activation strategies. (2) Current bioinformatics tools like AntiSMASH and 2nFinder, while invaluable, still fail to predict all critical genes or enzymes with novel functions. (3) Gene manipulation in wild-type strains is hindered by difficulties in protoplast preparation, limiting genetic modification options. Overcoming these challenges requires the development of more robust bioinformatic tools, improved BGC activation methods, and advanced genetic techniques tailored to filamentous fungi. The rapid development and integration of technologies such as gene editing, directed evolution, artificial intelligence (AI), AlphaFold, *de novo* protein design, and synthetic biology provide unprecedented opportunities, significantly accelerating research and application in MFNPs (Atanasov et al., 2021). Bioinformatics and AI have further enabled the rational design, analysis, and modification of key biosynthetic genes for MFNPs production. The activation of silent BGCs, optimization of production conditions, and application of metabolic engineering to enhance MFNPs yields will be critical in advancing MFNPs discovery. Interdisciplinary approaches that bridge genomics, chemistry, and pharmacology will be essential for translating these findings into clinical applications. By overcoming the remaining challenges in genome mining, the full potential of marine fungi as a source of novel bioactive molecules can be realized, paving the way for the next generation of marine-derived therapeutics.
